# Repeatability and reproducibility of deep-learning-based liver volume and Couinaud segment volume measurement tool

**DOI:** 10.1007/s00261-021-03262-x

**Published:** 2021-10-04

**Authors:** Amirkasra Mojtahed, Luis Núñez, John Connell, Alessandro Fichera, Rowan Nicholls, Angela Barone, Mariana Marieiro, Anthony Puddu, Zobair Arya, Carlos Ferreira, Ged Ridgway, Matt Kelly, Hildo J. Lamb, Felipe Caseiro-Alves, J. Michael Brady, Rajarshi Banerjee

**Affiliations:** 1 Division of Abdominal Imaging, Massachusetts General Hospital, Harvard Medical School, Boston, MA USA; 2 Perspectum Ltd., Gemini One, 5520 John Smith Drive, Oxford, OX4 2LL UK; 3 Department of Radiology, Leiden University Medical Centre, Leiden, The Netherlands; 4 Universidade de Coimbra, Coimbra, Portugal

**Keywords:** Liver resection, Cirrhosis, Hepatectomy, Couinaud, Post-hepatectomy liver failure, Hepatic function

## Abstract

**Purpose:**

Volumetric and health assessment of the liver is crucial to avoid poor post-operative outcomes following liver resection surgery. No current methods allow for concurrent and accurate measurement of both Couinaud segmental volumes for future liver remnant estimation and liver health using non-invasive imaging. In this study, we demonstrate the accuracy and precision of segmental volume measurements using new medical software, Hepatica™.

**Methods:**

MRI scans from 48 volunteers from three previous studies were used in this analysis. Measurements obtained from Hepatica™ were compared with OsiriX. Time required per case with each software was also compared. The performance of technicians and experienced radiologists as well as the repeatability and reproducibility were compared using Bland–Altman plots and limits of agreement.

**Results:**

High levels of agreement and lower inter-operator variability for liver volume measurements were shown between Hepatica™ and existing methods for liver volumetry (mean Dice score 0.947 ± 0.010). A high consistency between technicians and experienced radiologists using the device for volumetry was shown (± 3.5% of total liver volume) as well as low inter-observer and intra-observer variability. Tight limits of agreement were shown between repeated Couinaud segment volume (+ 3.4% of whole liver), segmental liver fibroinflammation and segmental liver fat measurements in the same participant on the same scanner and between different scanners. An underestimation of whole-liver volume was observed between three non-reference scanners.

**Conclusion:**

Hepatica™ produces accurate and precise whole-liver and Couinaud segment volume and liver tissue characteristic measurements. Measurements are consistent between trained technicians and experienced radiologists.

**Graphic abstract:**

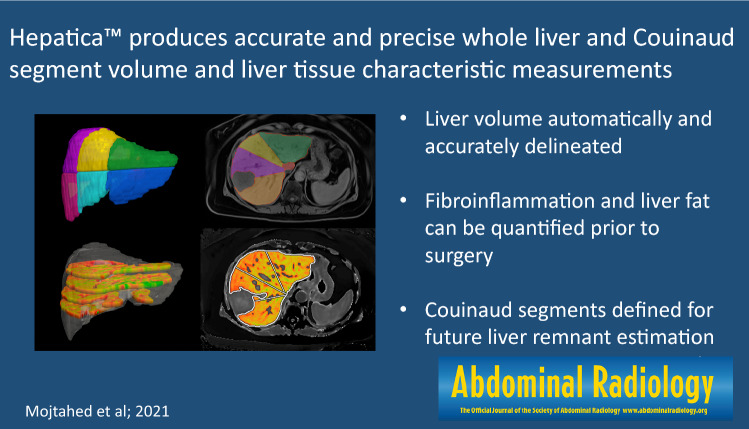

**Supplementary Information:**

The online version contains supplementary material available at 10.1007/s00261-021-03262-x.

## Introduction

Hepatocellular carcinoma is the fastest growing cause of cancer-related deaths in the USA [1], with 18,000 men and 9000 women dying each year. Metastasis to the liver remains a substantial problem [2] with 50,000 deaths each year [3, 4]. Guidelines from AASLD [5], EASL [6] and APASL [7] for management of primary liver cancer are generally congruous and patient outcomes are consistently most favourable when early-stage tumours are treated with surgical resection. Similarly for secondary liver cancer, surgical resection [8] currently represents the main curative therapy, often preceded by neoadjuvant chemotherapy or regional radiotherapy in order to suppress the spread and growth of the tumour. However, chemotherapy-associated steatohepatitis has implications for the functional reserve of the liver in patients undergoing surgery [9].

It is widely understood that the estimation of the volume and health of remnant liver parenchyma [10, 11] is a key parameter in assessing a resection plan. Underestimation of these factors can lead to post-operative liver failure [12–15]. As such, consensus opinion amongst surgeons is converging on agreement that a safe lower limit for the future liver remnant (FLR) should be 20% for a patient with a normal healthy liver parenchyma, 30% those with steatosis and 40% when liver has fibrosis or cirrhosis [16]. There is a lack of clear evidence guiding the modulation of safe lower FLR limits for an individual patient, often due to the failure to detect parenchymal liver disease prior to surgery. Interpreting liver function tests can be complex [17] and biopsy-derived pathology scores are associated with risk of haemorrhage and prone to sampling bias [18].

Formal anatomical resection techniques are widely applied to patients with multiple, or deep-lying, lesions such as the extended right hepatectomy where Couinaud segments 1, 4, 5, 6, 7 and 8 are removed. This often results in an FLR of 25–30%, indicating an unsuitability for patients with an inflamed or cirrhotic liver [19]. In such cases, alternative treatment management steps may be considered including two-stage processes such as pre-operative portal vein embolization or associating liver partition and portal vein ligation for staged hepatectomy (ALPPS), where hypertrophy of the FLR is encouraged and has been shown to improve safety of major hepatectomy procedures [20–25]. These complex strategies are being adopted more widely to reduce the risk of post-operative morbidity and long hospital stays [26].

Liver volume measurements and subsequent FLR estimations are typically performed by the radiologist using time-consuming manual techniques (up to 40 min/case [27, 28]) with associated intra-operator variability [28, 29]. In this study, we evaluate and assess performance of new medical software (Hepatica™), a medical device which performs automatic liver volumetry followed by semi-automatic delineation of the Couinaud segments, in comparison to clinical gold standard of experienced radiologists with a specialty in hepatic imaging. In addition to volumetry, the software tool reports validated biomarkers of liver health corrected T1 (cT1) and proton-density fat fraction (PDFF) [30], increasing the available information in pre-operative liver assessment to improve surgical decision-making.

## Methods

### Patients

Participants were selected amongst volunteers who had taken part in two previous ethically approved studies with informed consent. Liver MRI data of 48 volunteers from 2 different studies were used in evaluating the performance of the device. 18 participants (Group A) were used to verify the volumetry accuracy. 30 healthy volunteers (Group B) were used to evaluate the repeatability, reproducibility and intra- and inter-operator variability (See Supplementary Material for further details). These studies were approved by their respective ethics committee (IRAS IDs 241312 and 226607). Subject demographics are shown in Table 1. Data used in this analysis had not previously been used as training/test data for algorithmic validation or any other development of the device.

**Table 1:** Participant demographics of datasets used in Hepatica™ performance testing

	Group A	Group B	All groups
*n*	18	30	48
Sex (male:female)	11:07	11:19	22:26
Age (mean (min–max))	35 (24–64)	37 (18–60)	36 (18–64)
Reported healthy	13	20	33
AIH, PBC, PSC	5	4	9
Fatty liver	0	6	6

*PSC* primary sclerosing cholangitis, *AIH* autoimmune hepatitis, *PBC* primary biliary cirrhosis

### Image acquisition

Multiple scanners were used in this study in order to evaluate the reproducibility (same participant, different scanner) and repeatability (same participant, same scanner) of the new device. These scanners were Siemens Prisma 3T, Siemens Avanto fit 1.5T, GE Discovery MR750 3T, GE Optima MR450w 1.5T, Philips Achieva dStream 3T and Philips Ingenia 1.5T. Fat-saturated T1-weighted gradient recalled echo (GRE) images without contrast-agent were used for volumetric analysis. cT1, PDFF and T2* maps were acquired as previously described using multislice shortened MOLLI and IDEAL sequences [31, 32] with extensive validation. Imaging data as DICOM files are transferred using a secure online portal for analysis by an operator. Summary results are then reviewed and returned to the referring clinician as a report.

### Liver volume delineation

Hepatica™ delineates the liver from a 3D T1-weighted MR image. The volume corresponding to the liver is segmented using a convolutional neural network (CNN) that automatically delineates the liver including the caudate. A technician can then refine the volumes obtained from the CNN with manual edits using paintbrush tools and liver lesions are excluded from the segmentation and quantifications. Liver volume data were also analysed by two trained radiologists (9 and 12 years training) using OsiriX software.

### Couinaud segmentation

**Fig. 1:**
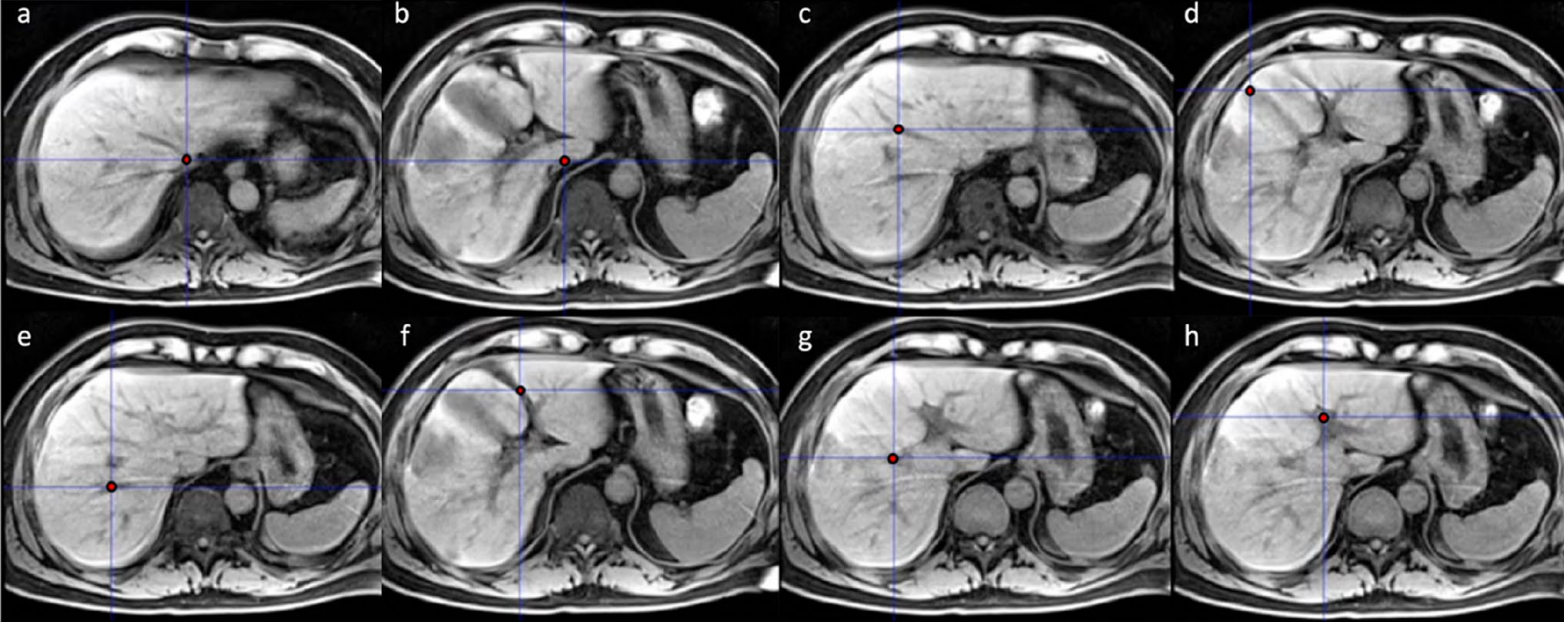
Anatomical landmarks used to delineate Couinaud segments: **a** Inferior vena cava (superior), **b** inferior vena cava (inferior), **c** middle hepatic vein, **d** gallbladder fossa, **e** right hepatic vein, **f** umbilical fissure, **g** right portal vein, **h** left portal vein

Couinaud classification of liver anatomy divides the organ into nine segments, based on the vasculature [33]. In the new device, a technician positions the following eight landmark points in an interactive 3D visualisation: inferior vena cava (superior zone), inferior vena cava (inferior zone), middle hepatic vein, gallbladder fossa, right hepatic vein, umbilical fissure, right portal vein and left portal vein (Fig. 1). Combinations of these landmarks are then used by Hepatica™ to define the planes that divide the liver into Couinaud segments.

### Quantitative liver tissue characteristics: cT1 and PDFF

Multislice cT1 extracted from Liver*MultiScan*™ (Perspectum Ltd., UK) has been demonstrated to be an accurate biomarker of hepatic fibroinflammation [34] in MR imaging and its combination with PDFF allows objective evaluation of future liver health. The multislice PDFF and cT1 data are then aligned with the volumetric MRI data to report the liver tissue characteristics within the volume of each individual Couinaud segment.

### Imaging parameters

cT1 maps are generated from 5 axial slices of T1 maps (shMOLLI) 8 mm thick with 12 mm gap, corrected for the presence of hepatic iron from a T2* map (DIXON) [35]. PDFF is measured from 5 × 20-mm-thick slices from IDEAL acquisition [32]. 3D T1-weighted images use vendor standard sequences within a single expiratory breath-hold, typically with a reconstructed resolution of 1.2 × 1.2 × 3.0 mm.

### Repeatability, reproducibility and intra/inter-operator variability

Comparisons between pairs of measurements were assessed using Bland–Altman plots, and 95% limits of agreement (LOA) were calculated. The accuracy of Hepatica™ reporting whole-liver volumetry was evaluated in comparison with OsiriX, both operated by two trained radiologists. Additionally, the accuracy of a trained technician reporting whole-liver and Couinaud segment volumetry, PDFF and cT1 was evaluated by comparing results of a technician with the average of two experienced radiologists. Time spent per case by users in both devices was also measured.

Precision is defined in terms of repeatability and reproducibility. Reproducibility is the difference of metrics of the same patient between the reference scanner (Siemens 3T Prisma scanner, selected as de facto reference scanner owing to availability) and non-reference scanners (Siemens Avanto fit 1.5T, GE Discovery MR750 3T, GE Optima MR450w 1.5T, Philips Achieva dStream 3T and Philips Ingenia 1.5T). Repeatability, performed on each of the six scanners, was measured as the difference between two acquisitions of the same patient under the same scanner, roughly 10 min apart. The patient was scanned, removed from the scanner, then returned and rescanned in order to induce realistic positional variation.

Intra- and inter-operator variability was assessed by one technician examining the same dataset twice and two technicians examining the same dataset.

## Results

### Accuracy of device compared to current gold standard

**Fig. 2:**
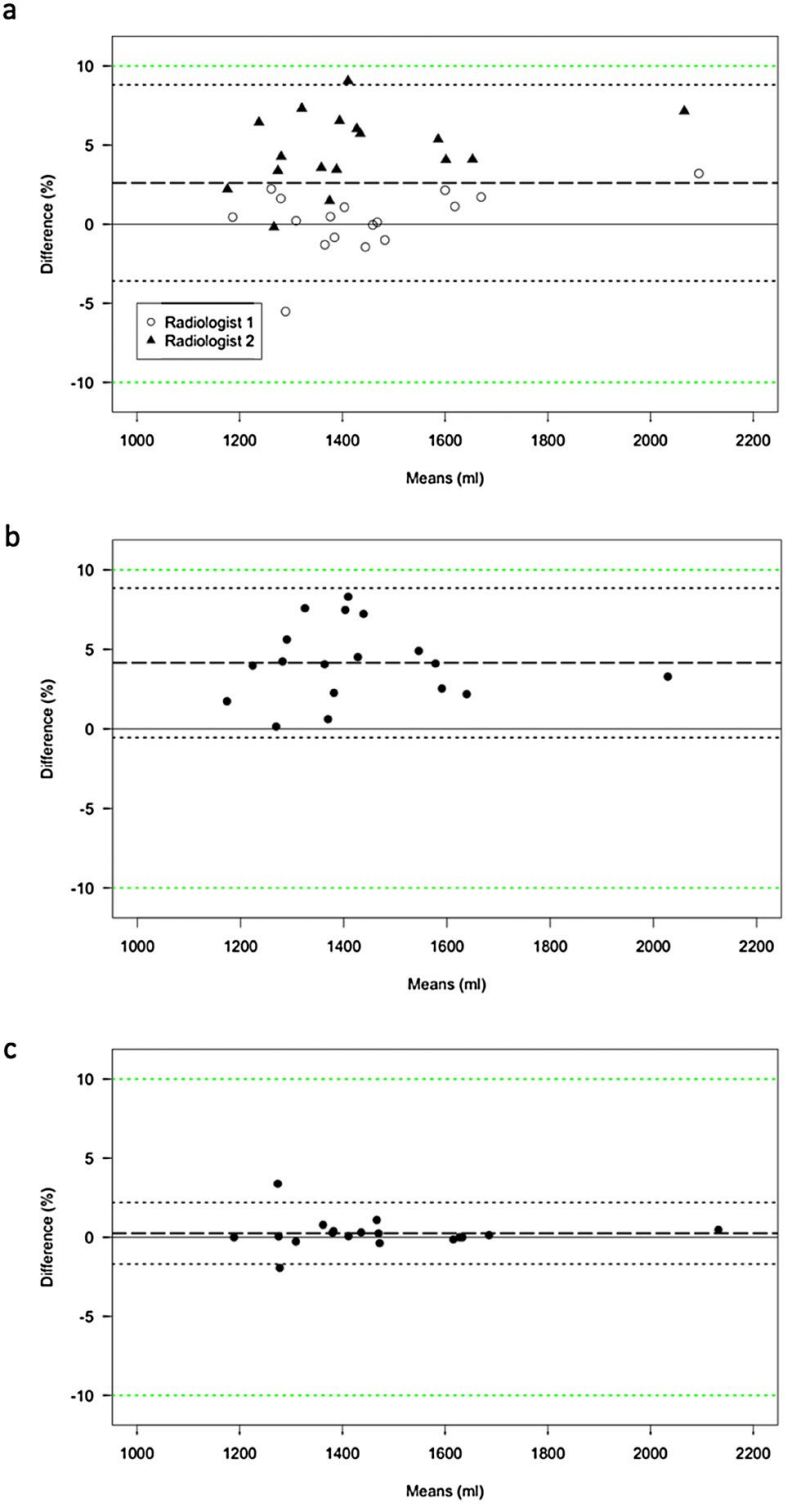
Comparison of volumetry between Hepatica™ and OsiriX. **a** Bland–Altman plot demonstrating agreement between volumetry by Hepatica™ and volumetry by OsiriX differentiating the two radiologists. Bias (dashed line) = 2.6%, LOA (black dotted line) [− 3.6%, 8.8%]. **b** Bland–Altman plot of variability between radiologists in volumetry using OsiriX, Bias (dashed line) = 4.2%, LOA (black dotted line) [− 0.5%, 8.9%]. **c** Bland–Altman plot of variability between radiologists in volumetry using Hepatica™, Bias (dashed line) = 0.2%, LOA (black dotted line) [− 1.7%, 2.2%]. Green dotted line = [− 10%, 10%]

**Fig. 3:**
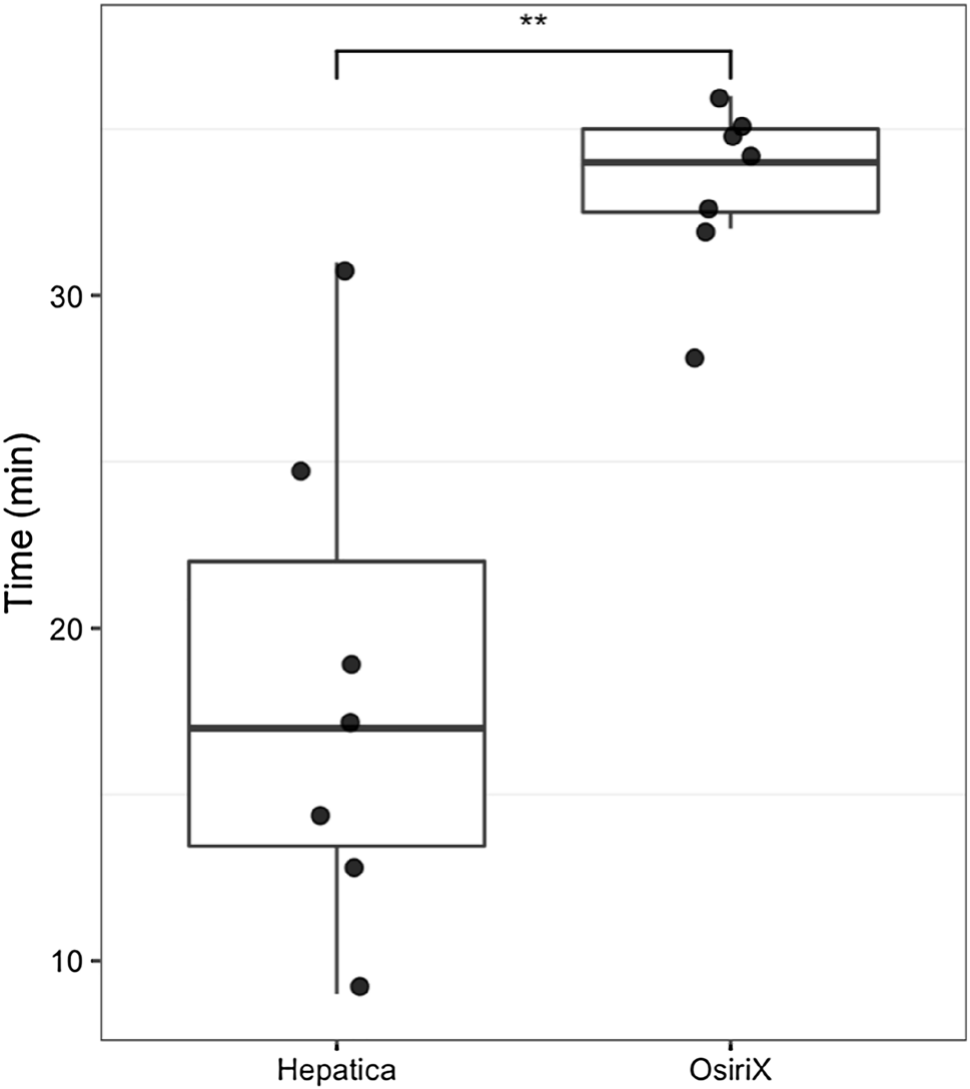
Time spent by one radiologist using software tools for whole-liver volumetry. *n* = 7 **p = 0.0033 Wilcoxon test

The similarity of whole-liver segmentations from two experienced radiologists using Hepatica™ and OsiriX was very high (*n* = 36 cases, 18 patients analysed separately by each radiologist, mean Dice score 0.947 ± 0.010), with resultant volume measurements from the two devices in strong agreement (Fig. 2a) across a range of typical liver volumes with very narrow upper and lower limits of agreement (LOA(%) = [− 3,6, 8.8]) of total liver volume. Hepatica™ showed higher agreement between two radiologists (LOA(%) = [− 1.7, 2.2]) compared to OsiriX (LOA(%) = [− 0.5, 8.8]) (Fig. 2b, c). The time spent to generate the whole-liver segmentation masks is significantly shorter whilst using Hepatica™ (median of 17 min per case) compared to OsiriX (median of 34 min per case) (*n* = 7 matched cases, **p = 0.0033 Wilcoxon test, Fig. 3).

Trained technicians using the new medical device demonstrated consistently high agreement when compared directly with experienced radiologists, with an average segment variability of ± 3.5% and whole-liver volume LOA = [− 4.2%, 0.5%] (Table 2). Segmental cT1 and segmental PDFF were in high agreement between technician and radiologists (Table 3), with average segment volume LOA of ± 1.1% and ± 0.2%, respectively.

**Table 2:** Operator variability assessment (trained technician)

Metric	Operator vs radiologist	Operator 1	Operator 2	Inter-operator variability
		Intra-variability	Intra-variability	
*n*	18	10	10	10
Whole liver (%LOA)	[− 4.2, 0.5]	[− 0.8, 0.9]	[− 4.1, 3.0]	[− 5.5, 2.7]
Segment 1 (%LOA)	[− 0.5, 1.0]	[− 0.4, 0.2]	[− 0.2, 0.1]	[− 0.2, 0.2]
Segment 2 (%LOA)	[− 3.1, 5.1]	[− 1.5, 3.0]	[− 2.1, 2.0]	[− 2.3, 3.5]
Segment 3 (%LOA)	[− 5.0, 3.9]	[− 2.5, 1.6]	[− 1.9, 2.2]	[− 2.3, 1.9]
Segment 4a (%LOA)	[− 4.6, 4.3]	[− 1.0, 2.0]	[− 1.8, 1.4]	[− 1.9, 2.0]
Segment 4b (%LOA)	[− 5.5, 2.6]	[− 1.8, 1.7]	[− 1.1, 1.1]	[− 2.1, 1.3]
Segment 5 (%LOA)	[− 1.5, 3.4]	[− 5.8, 6.6]	[− 2.6, 6.9]	[− 4.6, 3.7]
Segment 6 (%LOA)	[− 4.3, 4.3]	[− 4.9, 4.6]	[− 6.6, 8.2]	[− 6.7, 5.3]
Segment 7 (%LOA)	[− 3.3, 1.8]	[− 6.2, 5.5]	[− 7.7, 3.5]	[− 3.5, 3.9]
Segment 8 (%LOA)	[− 3.9, 5.5]	[− 6.2, 5.2]	[− 9.7, 8.1]	[− 4.5, 6.3]
Average of all segments	± 3.5	± 3.4	± 3.7	± 3.1

Limits of agreement (%) of whole-liver and Couinaud segment volume

**Table 3:** Limit of agreement (%) of whole-liver and Couinaud segment median cT1 and median PDFF

Metric	cT1	PDFF
*n*	10	10
Whole liver	[0.0, 0.0]	[0.0, 0.0]
Segment 1 (%LOA)	[− 1.1, 0.6]	[− 0.3, 0.2]
Segment 2 (%LOA)	[− 2.4, 1.6]	[− 0.3, 0.4]
Segment 3 (%LOA)	[− 1.5, 1.3]	[− 0.2, 0.2]
Segment 4a (%LOA)	[− 0.8, 1.1]	[− 0.3, 0.2]
Segment 4b (%LOA)	[− 1.3, 1.1]	[− 0.2, 0.1]
Segment 5 (%LOA)	[− 1.1, 0.9]	[− 0.2, 0.2]
Segment 6 (%LOA)	[− 1.0, 0.8]	[− 0.2, 0.3]
Segment 7 (%LOA)	[− 0.9, 0.6]	[− 0.1, 0.2]
Segment 8 (%LOA)	[− 0.9, 1.1]	[− 0.2, 0.3]
Average of all segments	± 1.1	± 0.2

### Repeatability

The consistency of volumetric measurement results from the device on each of the six different scanners was evaluated with repeat scans of ten participants scanned on/off/on, simulating a follow-up visit with no volumetric change. High repeatability was observed on each scanner used, where the broadest LOA appeared in Philips 1.5T for whole-liver volumetry, equal to [− 7.9%, 4.8%] (Table 4). The average liver segment volume LOA was equal or within ± 3.4% for all the scanners.

**Table 4:** Within participant repeatability analysis

Scanner	Siemens 3T	Siemens 1.5T	GE 3T	GE 1.5T	Philips 3T	Philips 1.5T
*n*	10	10	10	10	10	10
Whole liver	[− 4.3, 1.4]	[− 7.0, 5.0]	[− 6.0, 5.0]	[− 4.5, 2.4]	[− 7.1, 4.0]	[− 7.9, 4.8]
Segment 1 (%LOA)	[− 0.2, 0.2]	[− 0.3, 0.3]	[− 1.1, 0.9]	[− 0.2, 0.4]	[− 0.8, 0.8]	[− 1.2, 0.8]
Segment 2 (%LOA)	[− 1.9, 2.1]	[− 2.0, 2.8]	[− 5.1, 3.9]	[− 3.5, 5.1]	[− 2.3, 2.1]	[− 2.9, 2.9]
Segment 3 (%LOA)	[− 2.3, 2.4]	[− 2.8, 2.0]	[− 2.2, 4.5]	[− 4.1, 3.1]	[− 1.2, 2.4]	[− 2.5, 3.5]
Segment 4a (%LOA)	[− 3.2, 4.4]	[− 1.4, 1.6]	[− 3.0, 1.6]	[− 2.4, 2.7]	[− 1.6, 1.5]	[− 2.6, 2.2]
Segment 4b (%LOA)	[− 1.4, 2.0]	[− 1.0, 0.7]	[− 1.4, 2.5]	[− 3.1, 2.7]	[− 1.0, 1.1]	[− 2.4, 2.1]
Segment 5 (%LOA)	[− 5.6, 7.8]	[− 3.2, 5.7]	[− 4.6, 4.0]	[− 3.0, 2.1]	[− 4.4, 1.4]	[− 3.2, 3.2]
Segment 6 (%LOA)	[− 5.8, 2.7]	[− 4.3, 3.5]	[− 1.6, 4.2]	[− 1.7, 2.1]	[− 2.3, 5.3]	[− 2.9, 3.6]
Segment 7 (%LOA)	[− 5.0, 2.7]	[− 3.9, 2.9]	[− 3.1, 3.1]	[− 2.3, 3.4]	[− 2.1, 4.2]	[− 2.3, 2.4]
Segment 8 (%LOA)	[− 5.8, 7.0]	[− 4.8, 4.4]	[− 4.2, 1.5]	[− 2.8, 1.5]	[− 6.2, 3.1]	[− 4.5, 3.8]
Average of all segments	± 3.4	± 2.6	± 2.9	± 2.6	± 2.4	± 2.7

Upper and lower limits of agreement (%) of whole-liver and Couinaud segment volume

### Reproducibility

Volumetric measurements from a Siemens 3T scanner were compared against five other major scanner models and field strengths to measure variability amongst MRI scanner type (Table 5). No significant bias or variation was observed in any Couinaud segment relative volume between the reference scanner and five non-reference scanners. However, in GE Discovery MR750 3T, Philips Achieva dStream 3T and Philips Ingenia 1.5T, a whole-liver volume underestimation bias (LOA(%) = [− 14.0, 5.7], [− 22.9, 2.2] and [− 19.2, 6.2], respectively). Whole-liver volume measurements from Siemens Avanto fit 1.5T and GE Optima MR450w 1.5T had tighter LOAs(%) when compared to the reference scanner [− 7.2, 6.5] and [− 9.8, 4.2], respectively. All the scanners had an average segment volume LOA equal to or within ± 3.8%

**Table 5:** Between scanner reproducibility analysis

Reference scanner vs scanner	Siemens 3T vs Siemens 1.5T	Siemens 3T vs GE 3T	Siemens 3T vs GE 1.5T	Siemens 3T vs Philips 3T	Siemens 3T vs Philips 1.5T
*n*	10	10	10	10	10
Whole liver	[− 7.2, 6.5]	[− 14.0, 5.7]	[− 9.8, 4.2]	[− 22.9, 2.2]	[− 19.2, 6.2]
Segment 1 (%LOA)	[− 0.3, 0.3]	[− 1.0, 0.9]	[− 0.3, 0.3]	[− 2.1, 0.8]	[− 2.0, 1.1]
Segment 2 (%LOA)	[− 0.9, 2.4]	[− 1.9, 3.1]	[− 2.8, 3.9]	[− 4.3, 2.6]	[− 3.9, 2.0]
Segment 3 (%LOA)	[− 2.2, 1.4]	[− 2.9, 2.2]	[− 2.1, 1.1]	[− 2.8, 2.5]	[− 1.5, 2.7]
Segment 4a (%LOA)	[− 2.5, 2.3]	[− 2.8, 2.3]	[− 1.9, 2.0]	[− 3.7, 4.0]	[− 1.8, 2.2]
Segment 4b (%LOA)	[− 1.5, 1.4]	[− 1.9, 1.7]	[− 2.0, 2.1]	[− 1.5, 1.5]	[− 1.8, 2.2]
Segment 5 (%LOA)	[− 5.5, 8.0]	[− 4.8, 4.2]	[− 6.1, 7.5]	[− 5.8, 5.1]	[− 2.1, 4.4]
Segment 6 (%LOA)	[− 3.6, 6.1]	[− 1.2, 3.9]	[− 5.0, 6.6]	[− 3.9, 6.4]	[− 3.9, 2.9]
Segment 7 (%LOA)	[− 5.8, 3.9]	[− 3.1, 2.9]	[− 4.7, 3.6]	[− 4.3, 5.2]	[− 3.8, 1.9]
Segment 8 (%LOA)	[− 8.3, 4.8]	[− 2.7, 1.2]	[− 9.4, 7.1]	[− 5.5, 5.5]	[− 2.8, 4.3]
Average of all segments	± 3.4	± 2.5	± 3.8	± 3.7	± 2.6

Upper and lower limits of agreement (%) of whole-liver and Couinaud segment volume

### Intra- and inter-operator variability

Inter-operator, intra-operator 1 and intra-operator 2 whole-liver volumetry LOA were [− 0.8%, 0.9%], [− 4.1%, 3%] and [− 5.5%, 2.7%], respectively, and the average segment volume LOA was equal to or within ± 3.7% for all of them.

## Discussion

The aim of this study was to evaluate the accuracy, reproducibility, repeatability, intra- and inter-observer variability and time saving resulting from using Hepatica™ in relation to currently available software. Results indicate that the new medical device is capable of delineating livers accurately and of dividing the volume into Couinaud segments based on anatomical landmarks. This is performed with a substantial reduction in time compared with the current gold standard, which uses manual segmentation by an experienced radiologist. The automation of several steps results in reduced subjectivity and increased robustness of volumetry as shown by the reduction in variability of liver volumetry.

Robustness of this medical software was demonstrated in three aspects: Firstly, within the repeatability study, where despite the participant exiting the scanner and returning for a second scan, volumetric measurements were remarkably consistent, as demonstrated on six major MRI scanner models. Secondly, Couinaud segment volume measurements were also demonstrated to be highly reproducible when the same participant was subsequently scanned on separate scanner models. Finally, inter- and intra-operator variability was demonstrated to be low indicating that results are not impacted by subjective bias. Liver volumetry has previously been extensively examined in the context of liver resection surgery, transplant surgery and image analysis techniques; this is the first report of a highly robust deep-learning-based methodology that could be widely deployed using less experienced professionals to obtain accurate outputs.

Reproducibility tests for GE Discovery MR750 3T, Philips Achieva dStream 3T and Philips Ingenia 1.5T showed a higher disagreement compared to the reference scanner. These disagreements may stem from differences in contrast generated by different scanner models at the boundary of the liver. This effect may be consistent with any currently available volumetry software, but we are currently unaware of any between-scanner reproducibility studies in liver volumetry imaging.

**Fig. 4:**
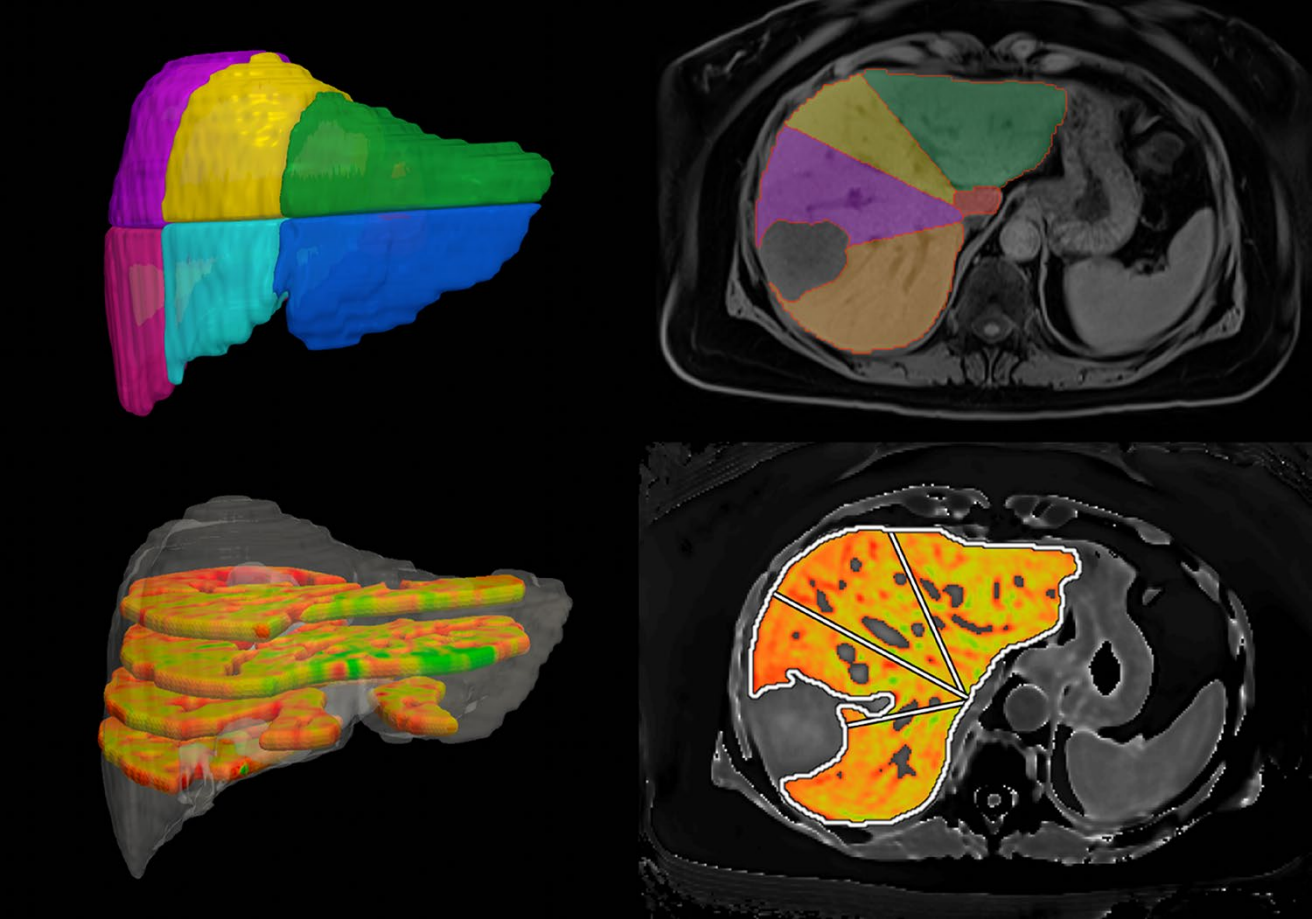
Example of Couinaud segment delineations from Hepatica™ on non-contrast-enhanced T1-weighted MR and cT1 images

The clinical utility of this medical software for preoperative planning is further demonstrated beyond saving time, as it quantifies liver health using cT1 and PDFF within the whole liver as well as within the remaining segments of the future liver remnant (FLR). Additionally, when the FLR of a particular operation is requested, radiologists must typically delineate each surgical option manually; in the device examined here, the volume of each Couinaud segment is generated (Fig. 4); thus, the FLR of each possible surgical option can be quickly measured (e.g. right hepatectomy versus segmentectomy of segment 5 and 8). Study limitations include the relatively small sample size (*n* = 48) and the associated low number of cases with underlying liver disease (*n* = 15). With the continued use of this device in more patients being considered for liver surgery, we intend to surveil the accuracy of liver tissue characterisation.

In conclusion, Hepatica™ is a new medical device that provides robust whole-liver and Couinaud segment volume and liver tissue characteristic measurements to support the treatment decision-making process. This enables surgeons to make individual assessments of a patient based on the volume and health of remnant livers prior to resection for liver cancer.

## Supplementary Information

Below is the link to the electronic supplementary material.

Supplementary file1 (DOCX 60 kb)
