# Supplementary material for: Repeatability and reproducibility of deep-learning-based liver volume and Couinaud segment volume measurement tool
Source: Abdom Radiol (NY). 2021 Oct 4;47(1):143–51. doi: 10.1007/s00261-021-03262-x (PMC8776724; doi:10.1007/s00261-021-03262-x)
Supplement: Supplementary file 1 — Supplementary file1 (DOCX 60 kb) [file 261_2021_3262_MOESM1_ESM.docx]

**Supplementary Materials:**

***Repeatability and reproducibility of deep-learning based liver volume and Couinaud segment volume measurement tool***

# **Distribution of patients for repeatability, reproducibility, intra-reader and inter-reader agreement tests**

In the next figure is represented how the 30 patients from Group B were distributed among scanners to be able to perform all the different tests, to make easier to the reader to understand the split of that cohort.
